# The utility of age-specific cut-offs for visual rating of medial temporal atrophy in classifying Alzheimer's disease, MCI and cognitively normal elderly subjects

**DOI:** 10.3389/fnagi.2013.00047

**Published:** 2013-09-18

**Authors:** Ranjan Duara, David A. Loewenstein, Qian Shen, Warren Barker, Daniel Varon, Maria T. Greig, Rosie Curiel, Joscelyn Agron, Isael Santos, Huntington Potter

**Affiliations:** ^1^Wien Center for Alzheimer's Disease and Memory Disorders, Mount Sinai Medical CenterMiami Beach, FL, USA; ^2^Departments of Medicine and Neurology, Miller School of Medicine, University of MiamiMiami, FL, USA; ^3^Department of Psychiatry and Behavioral Sciences, Miller School of Medicine, University of MiamiMiami, USA; ^4^Department of Neurology, Florida International University College of MedicineMiami, FL, USA; ^5^Departments of Molecular Medicine and Neurology, University of South FloridaTampa, FL, USA; ^6^Johnnie B. Byrd, Sr. Alzheimer's Center and Research InstituteTampa, FL, USA; ^7^Department of Biomedical Engineering, University of MiamiCoral Gables, FL, USA

**Keywords:** volumetric measures, hippocampus, visual rating, medial temporal atrophy, aMCI, Alzheimer's disease

## Abstract

**Background:** New research criteria for diagnosing Alzheimer's disease (AD) in the mild cognitive impairment stage (MCI-AD) incorporate biomarkers to assign a level of certainty to the diagnosis. Structural MRI is widely available but greatly under-utilized for assessing atrophy of structures affected in early AD, such as the hippocampus (HP), because the quantification of HP volumes (HP-v) requires special expertise, and normative values have not been established.

**Methods:** Elderly subjects (*n* =273) from the Florida ADRC were classified as having no cognitive impairment (cognitively normal, CN), amnestic mild cognitive impairment (aMCI) or AD. Volumes for the hippocampus (HP-v) were measured on structural MRI scans. A validated visual rating system for measuring medial temporal atrophy (VRS-MTA), including hippocampal, entorhinal cortex and perirhinal cortex atrophy was employed. The participants were subdivided into younger (less than or equal to 75 years of age) and older (greater than 75 years of age) subgroups.

**Results:** Volumetric and VRS-MTA measures were equivalent in predicting classification of CN vs. aMCI for older (area under the receiver operator curves [aROC]: 0.652 vs. 0.723) and younger subjects (aROC: 0.764 vs. 0.736). However, for younger AD subjects, aROC values were significantly higher for VRS-MTA measures (0.920) than for volumetric measures (0.847). Relative to HP-v, VRS-MTA score was significantly more correlated to impairment on a range of memory tests and was more associated with progression of aMCI to AD than HP-v.

**Conclusion:** Structural MRI with VRS-MTA assessment can serve as a biomarker for supporting the diagnosis of MCI-AD. Age-adjusted VRS-MTA scores are at least as effective as HP-v for distinguishing aMCI and AD from CN and for predicting progression from aMCI to AD. VRS-MTA is convenient for use in the clinic as well as for clinical trials and can readily be incorporated into a standardized radiological report.

## Introduction

Recently revised criteria for diagnosing an early clinical stage of AD (“Mild Cognitive Impairment, or MCI, due to AD”; MCI-AD) (Albert et al., 2011) and “Prodromal AD” (Sperling et al., 2011) incorporate biomarkers to increase the certainty of the diagnosis. One such biomarker, atrophy of the hippocampus (HP) and other medial temporal lobe (MTL) structures on structural MRI, increase the likelihood of a neurodegenerative disorder, such as AD, as the cause of MCI. In spite of the widespread use of MRI scans for the assessment of individuals with various forms of cognitive impairment, this biomarker is used primarily for excluding causes of cognitive impairment other than AD, such as hydrocephalus, vascular and space-occupying lesions. However, MRI can be used to confirm the presence of neurodegenerative pathology among patients presenting with MCI and dementia (Frisoni et al., 2010) and is greatly underutilized for this purpose by clinicians and radiologists. Although prodromal forms of AD are in a continuum with, and may be clinically indistinguishable from what is described as “Probable AD,” current diagnostic research standards incorporate a biomarker to support the diagnosis of prodromal AD or MCI-AD It is clear that the diagnosis of both Probable AD and Prodromal AD/MCI-AD would be more secure in the presence of a positive biomarker which provides further evidence of the presence of a neurodegenerative disease (Albert et al., 2011; Sperling et al., 2011).

Neurodegenerative changes such as atrophy which are characteristic of Alzheimer's disease (AD), and occasionally of other dementing diseases, such as Fronto-temporal Lobar Dementia (FTLD) or Hippocampal Sclerosis, may be detected using volumetric analysis or, more conveniently, using visual rating of MRI scans. Nevertheless, the incorporation of MRI for confirming the diagnosis of neurodegenerative disease has yet to receive widespread utility, in part because of (a) lack of awareness of the value and accuracy of MRI for this purpose, (b) automated, quantitative volumetric methods for measuring hippocampal volume are unwieldy, expensive and not easily adapted for routine clinical use, and (c) the lack of widely accepted age-adjusted norms and cut-scores for hippocampal volume (HP-v) and medial temporal atrophy (MTA).

The goal of this study was to evaluate user-friendly methods to evaluate structural MRI scans and to provide appropriate age-adjusted cut scores for both visually rated MTA measures and HP-v structures, which best distinguish normal elderly subjects from those who have AD. Accordingly, we compared hippocampal volumes (HP-v) to a refinement of the semiquantitative visual rating method, initially developed by Scheltens et al. (1995). This new visual rating system for assessing medial temporal atrophy (VRS-MTA) (Duara et al., 2008; Urs et al., 2009) provides a total MTA score by combining atrophy levels in individual medial temporal structures, including the HP, the entorhinal cortex (ERC), and the perirhinal cortex (PRC). We established appropriate age-related cut-offs for both volumetric measures of the HP and VRS-MTA measures, which correctly classified 70–80% of cognitively normal [CN] subjects without cognitive impairment. We chose these levels of specificity because at least 20–30% of CN subjects are known to harbor the pathology of AD on post-mortem evaluation (Morris, 2006). We then compared the accuracy of these two methods for distinguishing CN from subjects with aMCI and AD, the associations of these two measures with neuropsychological measures of cognition and the ability to predict progression from aMCI to dementia.

## Methods

### Subject recruitment

The current sample was recruited from a group of 273 subjects (107 CN, who were enrolled in the Florida Alzheimer's Disease Research Center Clinical Core (FADRC-CC) in Miami Beach FL between 2005 and 2009 (Duara et al., 2010). Subjects were diagnosed as cognitively normal (CN) or having amnestic MCI (aMCI) or dementia. The study was approved by the Institutional Review Board at Mount Sinai Medical Center, Miami Beach, and the University of South Florida, Tampa. All subjects or a legal representative provided informed consent.

### Evaluations

The following were completed on all subjects: (1) full clinical history, obtained from a reliable informant; (2) neurological evaluation; (3) psychiatric evaluation, including administration of the Geriatric Depression scale (Sheikh and Yesavage, 1986) and the Neuropsychiatric Inventory (Cummings et al., 1994); (4) Clinical Disease Rating scale (CDR-SB; Morris, 1993); (5) Mini-Mental State Examination (MMSE; Folstein et al., 1975); (6) a neuropsychological test battery, as described below; (7) Unified Parkinson Disease Rating Scale (UPDRS, motor section; Fahn and Elton, 1987) which has been documented as a sensitive tool for quantifying motor dysfunction and parkinsonism in patients with various forms of MCI and dementia.

Cardiovascular Risk (CVR) Score was calculated as the sum of 10 independent risk factors (14) selected from the National Alzheimer's Coordinating Center (NACC) Uniform Data Set (UDS) Subject Health History assessment protocol (Appel et al., 2009).

## Diagnostic procedures

### Determining a consensus diagnosis for cognitively normal, different MCI subtypes and dementia

The physician assigned a cognitive diagnosis of CN, MCI, or Dementia, as described previously (Duara et al., 2010). Briefly, the PhyDx was based on the subject's entire clinical history and functional status, which was derived from the history itself, CDR rating, functional activity questionnaire, MMSE score and sub-scores, taking into account the subjects' educational and cultural background, sensory (especially visual and hearing) and motor deficits, language and speech disorders, medical and psychiatric conditions and the perceived reliability of the informant. In addition to the physician's diagnosis, an independent neuropsychological diagnosis was rendered by a neuropsychologist.

### Neuropsychological diagnosis (NPDx)

All neuropsychological tests were administered in the subjects' native language (English or Spanish) and compared to age and education adjusted normative data, as described previously (Loewenstein et al., 2009). The tests included all of those outlined in the NACC protocol (Beekly et al., 2007), as well as additional tests, including the Three Trial Fuld Object Memory Evaluation (FOME; Fuld, 1981), and the Hopkins Verbal Learning Test-Delayed Recall (HVLT; Benedict et al., 1998). Memory measures were: the FOME, HVLT, and Delayed Visual Reproduction of the Wechsler Memory Scale-R (Wechsler, 1987). Non-memory tests included: category fluency (Monsch et al., 1992), letter fluency (language; Monsch et al., 1992), Block Design-WAIS-III (visuospatial; Wechsler, 1997), Trails B (Executive; Army Individual Test Battery, 1944), and Similarities-WAIS-R (Executive; Wechsler, 1997). Neuropsychological classification were made as follows: (a) a test score of 1.5 SD or greater below expected normative values on any single test for MCI syndromes; and (b) 2.0 SD or greater below expected normative values in one memory and one non-memory test for dementia (corresponding to NINCDS-ADRDA criteria; (McKhann et al., 1984). Nomenclature used for NPDx was Normal, Non-Amnestic MCI (naMCI; single or multi-domain), amnestic MCI (aMCI; single or multi-domain) and Dementia.

### Algorithmic consensus cognitive diagnoses (ALgDx)

An algorithmic approach to consensus diagnosis (Duara et al., 2010) combined the PhyDx with the NPDx, as follows: (a) a PhyDx and a NPDx of Normal received an AlgDx of cognitively normal (CN); (b) a PhyDx diagnosis of MCI and a NPDx of aMCI received an AlgDx of aMCI; (c) a PhyDx of dementia and a NPDx of aMCI or Dementia received an AlgDx of Dementia. Patients diagnosed with aMCI met Petersen criteria for MCI (Petersen et al., 1999). Probable AD was diagnosed according to National Institute of Neurological and Communicative Disorders and Stroke (NINCDS)–Alzheimer's Disease and Related Disorders Association (ADRDA) criteria for AD (McKhann et al., 1984) and the criteria set forth by the National Alzheimer's Coordinating Center.

MRI Scans were acquired using a proprietary 3-D volumetric protocol on a Siemens Symphony, 1.5 Tesla machine (Iselin, NJ) or a GE 1.5 T machine, using proprietary three-dimensional magnetization-prepared rapid-acquisition gradient echo (Siemens) or the three-dimensional spoiled gradient recalled echo (General Electric) sequences; MRI scans were acquired in the coronal plane, and contiguous slices with thickness of 1.5 mm or less were reconstructed.

### Volumetric analysis of brain MRIs

Volumetric analysis is performed using Individual Brain Atlas and Statistical Parametric Mapping (IBASPM; Alemán-Gómez et al., 2006). In IBAPSM, the volume of brain regions is calculated after normalization or spatial transformation to Montreal Neurological Institute (MNI; McGill University, 2009) templates. The scans are segmented into three types of tissue in each hemisphere: gray matter, white matter, and cerebrospinal fluid. An individual brain atlas for each subject is created with the transformation matrix obtained from the normalization step, and anatomical automatic labeling (AAL) to specify 116 regions. Hippocampal volume (HP-vol) was calculated as the ratio of the volume of each HP (right and left) to the total intracranial volume (Shen et al., 2011).

### Visual rating methods assessing brain MRIs

**Figure 1:**
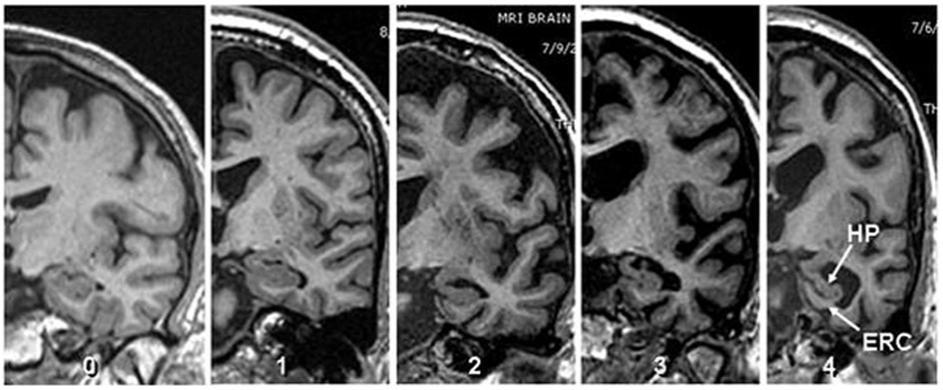
**Visual rating scale.** Image depicting four degrees of atrophy in Hippocampus and Entorhinal cortex according to visual rating scale where 0 = no atrophy, 1 = minimal atrophy, 2 = mild atrophy, 3 = moderate atrophy and 4 = server atrophy (Score shown corresponds to both structure).

The scope and utility of Scheltens' system was expanded by Duara et al. (2008) and Urs et al. (2009), to provide reliable visual ratings of individual MTL regions, i.e., hippocampus (HPC), ERC, and PRC. Reliability and accuracy were achieved using very thin coronal slices (1.2 to 1.5 mm thickness), perpendicular to the AC-PC line and intersecting the mammillary bodies (Urs et al., 2009). We have previously reported excellent inter-rater reliability for measuring individual MTL structures; kappa values among two raters ranged between 0.75 and 0.94 for inter-rater reliability and 0.87 and 0.93 for intra-rater reliability. (Urs et al., 2009). With VRS-MTA, semi-quantitative assessments of atrophy of the HP, ERC, and PRC were assigned as follows: a score of Grade 0 corresponded to no atrophy, Grade 1 to minimal atrophy, Grade 2 to mild atrophy, Grade 3 to moderate atrophy and Grade 4 to severe atrophy (Figure 1). The VRS-MTA program provides a library of drop-down images, depicting the anatomical boundaries of these structures as well as each grade of atrophy for the ERC, HP, and PRC.

ApoE genotype was determined using standard methods (Wenham et al., 1991). ApoEε 4 frequencies were subsequently calculated for each diagnostic group.

### Deriving cut-offs for HP-vol and VRS-MTA values for different age groups and both brain sides

To derive cut-scores for HP-vol (measured as percentage of intracranial volume) for the older CN group we used the scores of 20 subjects, aged 76 years and above (mean age = 79.64 years; *SD* = 3.2 years range = 76–90 years), who had an algorithmic diagnosis of CN. The cut-off scores for the lowest 20% (liberal cut score ≤0.0249%) and 30% (conservative cut score ≤0.0224%) of HP-vol, for each side, which was then used to identify hippocampal atrophy for all diagnostic groups aged 76 or greater. Similarly, we determined the approximate cut score (range of 0–12 points for each side), for highest 20–30% (liberal cut score ≥4) or 30–40% (conservative cut score ≥5) of combined HP, ERC, and PRC ratings on the left and right sides. These cut scores were then used to identify threshold levels of MTA for all diagnostic groups, aged 76 years or greater, separately for the right and left sides in each subject.

To derive a cut-score for the younger CN group, we took the scores of 87 cognitively normal individuals aged 63–75 years (mean age = 68.33 years; *SD* = 3.2 years) and used a similar procedure as for the older CN. The derived cut scores for HP-v (liberal cut score ≤0.027; conservative cut score ≤0.0257) and VRS-MTA ratings (liberal cut score ≥2.0; conservative cut score ≥3) for the right and left sides for each subject. In addition, so as to identify localized atrophy within the medial temporal region on each side, independent of the total VRS-MTA score, we determined the highest VRS scores for the right and left HP and ERC for each subject. For these measures a liberal (≤1.5) and a conservative (≤2.0) cut score were determined that would classify not more than 20 or 33% of both young and old CN group as having abnormal atrophy. These cut scores were also applied to subjects diagnosed with amnestic MCI and dementia.

### Longitudinal evaluation procedures

A total of 72 of the 103 subjects had at least one-annual follow-up evaluation (mean = 33.1 months; *SD* = 14.1 months), including neurological, psychiatric and neuropsychological evaluations, and re-diagnosis by the AlgDx. The mean age of this sample was 76.8 (*SD* = 5.8 years) and mean MMSE scores of were 26.1 (*SD* = 2.4) making the sample comparable to the aMCI patients who were originally diagnosed at baseline.

### Statistical analysis

Group comparisons of demographic variables across three study groups were analyzed using analyses of variance (ANOVA) or chi-square tests, as appropriate. *Post-hoc* tests of means were examined by the Tukey-Kramer procedure at *p* < 0.05. Comparative analyses of VRS-MTA and HP-v measures were assessed using receiver operator (ROC) curves. HP-v and MTA-VRS scores were correlated to a broad array of cognitive measures among memory impaired patients. Comparisons between correlation coefficients were tested statistically using SISA binomials (Uitenbroek, 1997). Finally, differences in progression rates across groups were assessed using chi-square procedures.

## Results

### Demographics

In the entire sample of subjects (age range = 63–93 years; mean age = 75.0 ± 7.2 years) there were statistically significant demographic differences between CN, aMCI, and AD groups, with regards to age, gender and educational attainment as well as on MMSE scores [*F*_(2, 269)_= 172.05; *p* < 0.001] (Table 1). *Post-hoc* tests revealed that CN patients were younger, better educated, had higher MMSE scores and were more frequently female compared to the other two groups. AD subjects were older and had lower MMSE scores than aMCI subjects. There were significant group differences with regards to Spanish vs. English-speaking subjects or percentage of subjects carrying one or more ApoEϵ4 allele. As indicated in Table 1, CN subjects scored higher than aMCI and AD subjects on all neuropsychological measures, and demonstrated less atrophy in comparison to aMCI and AD subjects on VRS-MTA and HP-v scores. AD subjects had more atrophy than aMCI subjects on VRS-MTA and HP-v measures, as well as impairment on all neuropsychological measures, with the exception of the Block Design test, in which there was no difference in scores between AD and aMCI subjects.

**Table 1:** **Demographics and MRI measures**.

	**CN (***n*** = **107**)**	**aMCI (***n*** = **105**)**	**AD (***n*** = **56**)**	***f*-value**
Age	71.1^c^ (5.8)	77.9^b^ (5.3)	79.5^a^ (6.8)	42.59^***^
Education	15.0^a^ (3.2)	12.4^b^ (3.9)	12.4^b^ (4.1)	16.71^***^
Gender (Female)	75.7%	50.0%	53.7%	X^2^= 16.93^***^
Hispanic%	47.4%	48.0%	57.4%	X^2^= 1.64^***^
ApoE%	24.7%	30.6%	42.9%	X^2^= 4.13 ^***^
MMSE	29.0^a^ (1.1)	25.9^b^ (2.5)	22.4^c^ (3.1)	172.05^***^
Fuld OME	25.7^a^ (2.0)	18.9^b^ (4.8)	10.6^c^ (6.5)	195.35^***^
HVLT-Total Recall	25.3^a^ (4.3)	17.4^b^ (4.5)	13.2^c^ (4.7)	159.48^***^
HVLT-DEL	9.2^a^ (1.7)	3.7^b^ (2.9)	1.3^c^ (2.2)	258.75^***^
Semantic interference test (SIT) score	13.3^a^ (2.9)	8.2^b^ (3.2)	3.1^c^(3.0)	209.04^***^
Visual reproduction test-delayed	23.0^a^ (7.9)	8.3^b^ (7.2)	3.4^c^ (5.7)	149.08^***^
Memory for Passages (Delayed)	11.5^a^ (3.5)	5.6^b^ (3.7)	2.2^c^ (3.0)	149.92^***^
Two Category Fluency	34.2^a^ (7.3)	24.1^b^ (6.2)	17.2^c^ (5.9)	134.24^***^
Block Design- WAIS-IV	31.5_a_ (9.4)	19.11^b^ (7.9)	18.8^b^ (7.8)	64.39^***^
Trails A	35.9^a^ (11.3)	54.5^b^ (23.9)	73.5^c^ (33.5)	55.04^***^
Trails B	95.1^a^ (48.2)	199.4^b^ (88.6)	254.1^c^ (73.7)	106.58^***^
HP-v (most impaired side)	0.00275^a^ (0.0003)	0.00240^b^ (0.004)	0.00208^c^ (0.005)	60.3^***^
VRS-MTA score (most impaired side)	1.7^a^ (1.8)	4.2^b^ (2.7)	6.9^c^ (3.3)	81.31^***^

*CN, Cognitively normal; aMCI, amnestic cognitive impairment; MMSE, Mini-mental status exam; HVLT, Hopkins verbal learning test; HP-v, Hippocampal volume; VRS-MTA, Visual rating scale-mdial temporal atrophy*.

*** *p < 0.001; means with different superscripts are statistically different at p < 0.05 by the Tukey-Kramer test*.

### Performance of VRS-MTA vs. HP-v

For the discrimination of amnestic MCI from CN, in both the younger and older age groups, there was no difference in the areas under receiver operating curve (aROC) between HP-v measures and VRS-MTA measures (*Z* = 1.26; *p* < 0.27) (Table 2). For the discrimination of AD from CN, among the younger age group (63–75 years), VRS-MTA performed better than HP-v (aROC: 0.92 vs. 0.847, *p* < 0.046). The corresponding sensitivity/specificity values for VRS-MAT and HP-v were: 89.2/82.1% and 75.7/82.1% for the more liberal cutoffs described in the methods. There was no difference between the performance of VRS-MTA and HP-v in the older group, for the classification of AD vs. CN. Considering the correct age-associated cut-offs for impairment for the total sample, 63% of those subjects who did not meet criteria for impairment using HP-v, did meet criteria for impairment using VRS-MTA, and conversely, only 30% of those who were VRS-MTA negative were HP-v positive.

**Table 2:** **HP-v and VRS-MTA measures in the classification of subjects with Amnestic MCI and Alzheimer's disease**.

**Diagnostic comparison and age group**	**Sensitivity/Specificity (%) for HP-v**	**aROC for HP-v measure**	**Sensitivity/Specificity (%) for VRS-MTA**	**aROC for VRS-MTA measure**	**Comparison of aROCs for Hp-v and VRS-MTA**
**AMNESTIC MILD COGNITIVE IMPAIRMENT VERSUS ELDERLY NORMAL**					
63–75 years (*n* = 60)	35.0/82.1	0.652 (*SE* = 0.06)	55.0/82.1	0.723 (*SE* = 0.06)	*Z* = 1.38; *p* > 0.26
76+ years (*n* = 45)	60.0/81.6	0.764 (*SE* = 0.05)	51.1 /78.2	0.736 (*SE* = 0.05)	*Z* = 0.60; *p* > 0.54
**ALZHEIMER'S DISEASE VERSUS ELDERLY NORMAL**					
63–75 years (*n* = 37)	75.7/82.1	0.847 (*SE* = 0.05)	89.2/82.1	0.920 (*SE* = 0.03)	*Z* = 2.00; *p* < 0.046
76+ years (*n* = 19)	63.2/81.6	0.713 (*SE* = 0.08)	68.4/78.2	0.853 (*SE* = 0.04)	*Z* = 1.66; *p* < 0.10

*HP-v, hippocampal volume; VRS-MTA, Visual rating system- medial temporal atrophy; aROC, Area under the receiver operating curve; AD, Alzheimer's disease; MCI, amnestic MCI; CN, Cognitively normal*.

### Correlations with cognitive measures

In a combined group of aMCI and AD subjects, who had adequate cognitive testing data, both HPv and VRS-MTA measures were strongly correlated with scores on various memory tests and with the category fluency test (a measure of speed of search from semantic lexicon) (Table 3). Tests of visuospatial function (block design), processing speed and attention (Trails A) and executive function (Trails B) were not correlated significantly with HP-v or VRS-MTA imaging measures. In most instances the memory measures (using the Fuld OME, for example) were more strongly correlated with VRS-MTA (*r* = −0.51) than with HP-v (*r* = −0.35) (*t* = 2.33; *p* < 0.02) (Table 3).

**Table 3:** **Comparative correlations between volumetric and VRS-MTA measures among 129 cognitively impaired patients**.

	**Correlation with HP-v**	**Correlation with VRS-MTA**	**Test of difference in correlations**	***p*-value coefficients^#^**
Fuld object memory evaluation	0.35^***^	−0.51^***^	2.33	<0.011
HVLT (Delayed Recall)	0.15	−0.29^***^	1.91	<0.030
WMS- memory for passages (Delayed Recall)	0.20^*^	−0.36^***^	2.27	<0.013
WMS-visual reproduction (Delay Recall)	0.33^***^	−0.47^***^	1.96	<0.027
Two word category fluency	0.31^***^	−0.32^***^	0.14	446
Trails A	−0.14	0.07	NA	NA
Trails B	−0.05	0.11	NA	NA
Block-design WAIS-II	0.05	−0.09	NA	NA
Similarities WAIS-R	−0.10	0.09	NA	NA

*HP-v, hippocampal volume; VRS-MTA, Visual rating system-medial temporal atrophy*.

# Difference in correlations tested using SISA polynomials (Uitenbroek, 1997)/

* p < 0.05,

*** *p < 0.001*.

### Progression from aMCI to AD

Among a sample of aMCI subjects (*n* = 72; mean age = 76.8 ± 5.8 years: educational attainment = 12.83 ± 3.6 years) with adequate follow-up data (mean follow-up period = 33.1 ± 14.1 months) the percentage of progressors vs. non-progressors to AD was predicted using both stringent and liberal VRS-MTA cut-off scores (Table 4). Using stringent VRS-MTA criteria, 51% of aMCI subjects scoring at or above the impairment cut-off were found to be progressors, as compared to 21% scoring at or above the impairment cut-off being non-progressors (χ^2^ = 5.51; *p* = 0.019). In contrast, for HP-v 41 % of aMCI subjects scoring at or above the impairment cut-off were found to be progressors, as compared to 29% scoring at or above the stringent impairment cut-offs being non-progressors (χ^2^ = 1.19; *p* = 0.28).

**Table 4:** **VRS-MTA score, HP-v and progression from aMCI to AD**.

	**Progressors to AD**	**Non-progressors**	**Chi-square**	***p-*value**
VRS-MTA score (Conservative criteria)	50.7%	20.6%	5.51	0.019
HP-v (Conservative criteria)	44.1%	28.9%	1.19	0.275

*HP-v, hippocampal volume; VRS-MTA, Visual rating system-medial temporal atrophy*.

## Discussion

In this study, we showed that VRS-MTA is superior to volumetric assessment of the HP (HP-v) for distinguishing aMCI patients and normal elderly controls. When we divided the subjects into “young-old” (63–75 years) and “older-old” (76 years+) subgroups, our previous findings hold true, in much smaller groups of subjects (Duara et al., 2008; Shen et al., 2011). We have also shown that VRS-MTA ratings correlate more strongly than do HP-v with memory measures and CDR ratings (Shen et al., 2011). In this study, we have additionally provided age-corrected cut-scores for HP-v and VRS–MTA scores for classifying subjects with aMCI and AD and have shown that VRS-MTA scores, but not HP-v scores in this cohort, were predictors of progression from aMCI to AD.

The use of structural MRI scans as biomarkers, in association with clinical criteria, for distinguishing CN subjects from those with incipient or Probable AD requires the use of age-adjusted cut- scores for research and for clinical practice. For the first time, to our knowledge, we have shown that specific age-related cut-scores for VRS-MTA and HP-v measures can be used for distinguishing CN from AD subjects. By deriving scores for the most impaired hemisphere, based upon age-related norms, we may have further enhanced the overall sensitivity of VRS-MTA. Indeed, almost two thirds of those subjects who did not meet criteria for impairment using HP-v, did meet criteria for impairment using VRS-MTA, and conversely, less than a third of those who were VRS-MTA negative were HP-v positive. Thus, each measure provides unique information, most notably VRS MTA, which includes independent and additive measures of the HP, ERC, and PRC.

An advantage of using VRS-MTA over HP-v is that regional brain volumes are variable across individuals and need to be normalized by conversion to a ratio of the absolute volume of the HP to intracranial volume, whereas VRS-MTA has built–in normalization and thus avoids multiplicative errors inherent in using ratios of two quantitative variables. From the clinician's vantage point, VRS-MTA has the following additional desirable attributes: (1) measurement and scoring of VRS-MTA is quick and reliable by the clinician, providing a distinct advantage over traditional volumetric techniques; (2) HP-v measurements, as compared to VRS-MTA measurements, require much greater technical stringency in the acquisition of the MRI scans and are far more vulnerable to a variety of measurement errors; (3) HP-v measurements require a technical interface for obtaining quantitative assessment whereas VRS-MTA does not (Duara et al., 2008; Shen et al., 2011). Although volumetric analysis of regional brain atrophy can be performed by a variety of programs which are widely available, they have been used almost exclusively in research applications, and not in clinical practice. Currently, measurement of HP-v is inconvenient and expensive in time and money and technical problems that often occur during the image acquisition protocol may invalidate the use of a substantial proportion of MRI scans performed in the community.

From a biological standpoint it is clear that hippocampal atrophy is non-specific and that the characteristic pathological changes in AD (Braak and Braak, 1985; Braak et al., 2006) begin outside the HP, with development of neurofibrillary tangles in the transentorhinal and entorhinal cortex, spreading subsequently to the subiculum and CA1 regions of the HP. Subsequent spread of pathology occurs to limbic, and ultimately to neocortical regions, such as the precuneus, middle frontal gyrus and posterior cingulate gyrus. The severity of this atrophy, at least in the medial temporal regions, correlates with the severity of underlying AD-related neuropathological changes seen on postmortem (Jack et al., 2002).

The use of VRS-MTA methodology affords a unique perspective, not available to those using quantitative HP-v measures, of the presence and severity of the neurodegenerative process in AD. Atrophy of the entorhinal and perirhinal cortices and the HP, widening of the collateral sulcus and atrophy of the white matter band between the subiculum and the ERC are well known pathological features of AD and are readily visible on appropriately obtained MRI scans acquired or reconstructed in the coronal plane in thin, contiguous brain slices. This information often times serves to confirm the clinical diagnosis, especially in a patient in which non-neurodegenerative causes of cognitive impairment, such as cerebrovascular disease or psychiatric conditions are also under consideration. The absence of confirmatory neurodegenerative findings on the MRI scan alerts the clinician to alternative causes of impaired cognitive performance, such as systemic disorders, attention deficit disorders, sleep-apnea syndrome, depression, anxiety, and cultural or language related factors.

The current investigation has the following advantages over previous studies: (1) Optimal age- related cut-scores for VRS-MTA and HP-v have been derived for normal subjects and then applied to aMCI and AD cases; (2) the importance of frequently-observed asymmetrical atrophy in medial temporal regions and HP-v volumes has been recognized and incorporated into the algorithm for distinguishing CN from aMCI and AD subjects, using either VRS-MTA or HP-v measures (typically, rather than using the most atrophic side in the algorithm, bilateral regions are combined into a single score). Using these methods our results indicate that VRS-MTA is at least as good, and more likely better than using HP-v for distinguishing both younger and older aMCI and AD subjects from CN subjects. VRS-MTA scores are also better correlated than are HP-v measures with memory and functional indices. Finally, VRS-MTA measures are better than HP-v measures in predicting progression to AD or dementia over a defined period of time. This suggests that VRS-MTA may provide a clearer indication of neurodegenerative pathology related to AD than merely HP-v.

Some of the limitations of using VRS-MTA include the fact that ratings are based on assessments performed on a single coronal slice, thereby providing a limited perspective of overall brain pathology (this limitation can be easily overcome by evaluating multiple adjacent coronal slices). In addition, atrophy in the medial temporal regions may not be specific to AD, but in some cases may be indicative of hippocampal sclerosis, frontotemporal lobar dementias, Lewy body dementia, vascular dementia, or cognitive impairment (Jack et al., 2002; Barkhof et al., 2007). Also, a larger and more diverse group of elderly normals will be required to extend age-related cut-off scores further than we have been able to do in this study. Age is a risk factor for AD and other neurodegenerative disorders and up to 30% elderly adults with underlying brain pathology may have sufficient cognitive reserve so that they do not present with cognitive symptoms. Hence, it is likely that among elderly volunteers, who are cognitively normal, substantial AD pathology is present, which may be reflected in their VRS-MTA scores, thereby apparently reducing the specificity of VRS-MTA cut-off scores.

At present, the primary utility of structural MRI, in the diagnosis of disorders causing cognitive impairment, is to rule out specific pathologies such as pathologies as hydrocephalus, vascular, inflammatory or demyelinating, and space-occupying lesions as the cause of the cognitive syndrome, but not for confirming the presence of AD-like pathology and its severity. Our results suggest that VRS-MTA, which could readily be incorporated into the routine assessment of patients presenting with memory symptoms, will likely assist in strengthening the diagnosis of AD or ruling it out, thereby improving both sensitivity and specificity of a clinical diagnosis of probable and prodromal AD. Moreover, VRS-MTA need not be used exclusively for clinical purposes; it could also serve as a research tool, especially in clinical trials when accuracy of the clinical diagnosis is a major requirement.

### Conflict of interest statement

The authors declare that the research was conducted in the absence of any commercial or financial relationships that could be construed as a potential conflict of interest.
